# Tunnel’ radicular cyst and its management with root canal treatment and periapical surgery: A case report

**DOI:** 10.1002/ccr3.2942

**Published:** 2020-05-25

**Authors:** Nik Rozainah Nik Abdul Ghani, Nor Faharina Abdul Hamid, Mohmed Isaqali Karobari

**Affiliations:** ^1^ Conservative Dentistry School of Dental Sciences Health Campus Universiti Sains Malaysia Kubang Kerian Malaysia; ^2^ Centre of Restorative Dentistry Studies Faculty of Dentistry Universiti Teknologi Mara Sungai Buloh Malaysia; ^3^ Conservative Unit School of Dental Sciences Health Campus Universiti Sains Malaysia Kubang Kerian Malaysia

**Keywords:** cell rest of malassez, periapical surgery, periodontal ligament, radicular cyst, root canal

## Abstract

An untreated root canal infection usually stimulates the development of a radicular cyst. Nonsurgical root canal procedures and periapical surgery followed by placement of bone substitute will promote the healing process of the bony defect.

## Graphical abstract

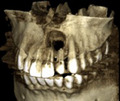

## 1 INTRODUCTION

Radicular cyst is formed by the stimulation and proliferation of an epithelial residues cell or rest of Malassez in the periodontal ligament. Tunnel or hollow appearance of radicular cyst occurred when both buccal and palatal cortical bone plates becoming lost or ruptured due to the extension of the cystic lesion.

Radicular cyst is a type of an inflammatory cyst in the jaw which is of odontogenic origin. It is formed by the stimulation and proliferation of epithelial residues (cell rests of Malassez) in the periodontal ligament, following dental trauma or caries.^1^ The cyst is commonly located at root apices of offending tooth or at the lateral side if associated with a lateral or accessory canal. A radicular cyst remains asymptomatic and the patient usually becomes aware of the cyst when a swelling becomes clinically obvious. Conventional orthograde root canal treatment is the regular approach for the management of the cyst. However, marsupialization, decompression, and surgical enucleation may be required for the cyst with a large and extensive size.^2^ This case report describes a tunnel radicular cyst (through‐and‐through cyst) of the anterior maxilla with clinical presentation of a labial mucosa swelling which was successfully treated by conventional root canal treatment, cyst enucleation, and placement of bone graft at the surrounding bone defect.

## 2 CASE PRESENTATION

**Figure 1:**
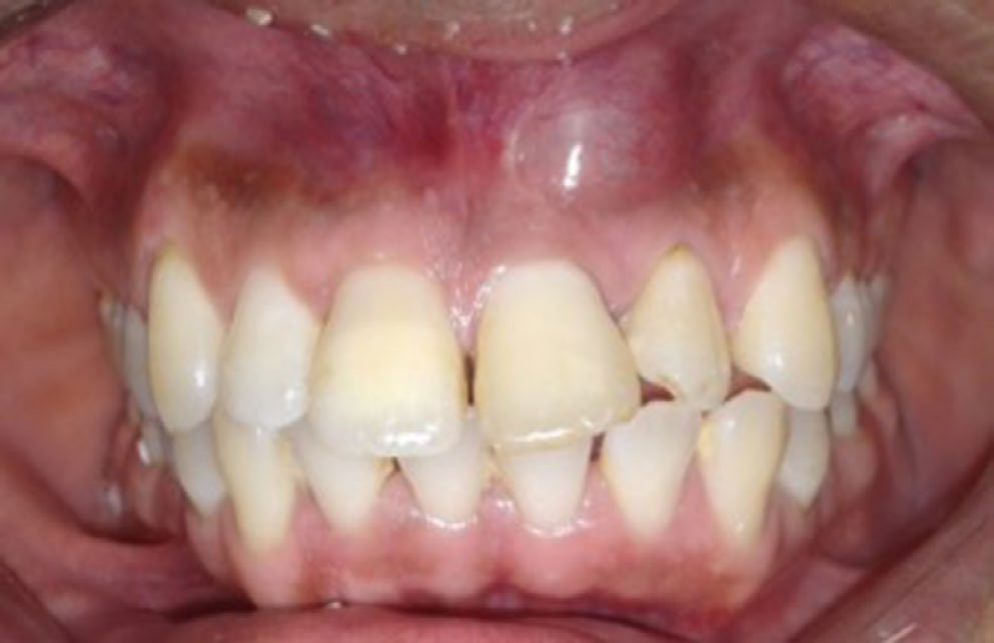
Flunctuant of labial mucosa on the area of upper left central incisor and upper left lateral incisor

A 31‐year‐old man was referred to the postgraduate endodontic clinic, regarding a considerable swelling on the buccal area of upper left central incisor and upper left lateral incisor. The patient presented with a history of trauma since 5 years, where a hard object knocked the upper anterior teeth. The teeth had never experienced any pain until recently, where he noticed the painless swelling, which started to increase in size and gave him discomfort. There was no significant medical illness, but the patient reported with allergy to Penicillin. Intraoral examination revealed a fluctuant swelling, around 2 × 2 cm on the labial mucosa from the area of upper left central incisor to the upper left lateral incisor. It was soft, nonmobile, nontender, and had a normal gingival color (Figure 1). The teeth showed no signs of mobility but were slightly tender to percussion. The patient presented with negative responses to thermal and electronic pulp tests in relation to the upper right central incisor, upper left central incisor, and upper left lateral incisor. The other anterior teeth had normal responses.

**Figure 2:**
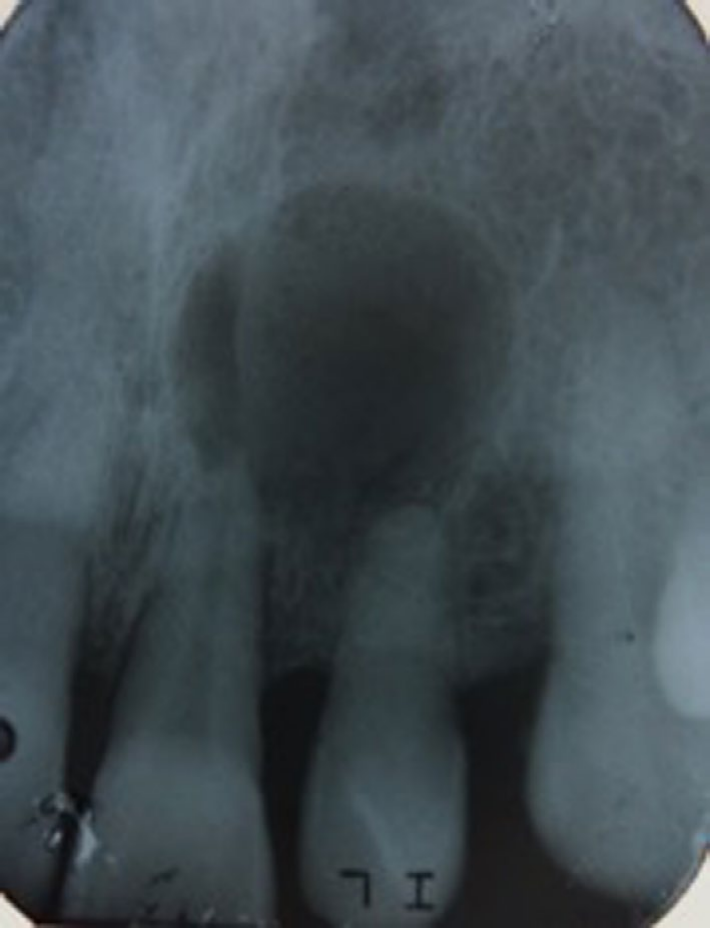
Periapical radiograph showing 2 × 2 cm well‐circumscribed radiolucent lesion involving apices of upper left central incisor and upper left lateral incisor

**Figure 3:**
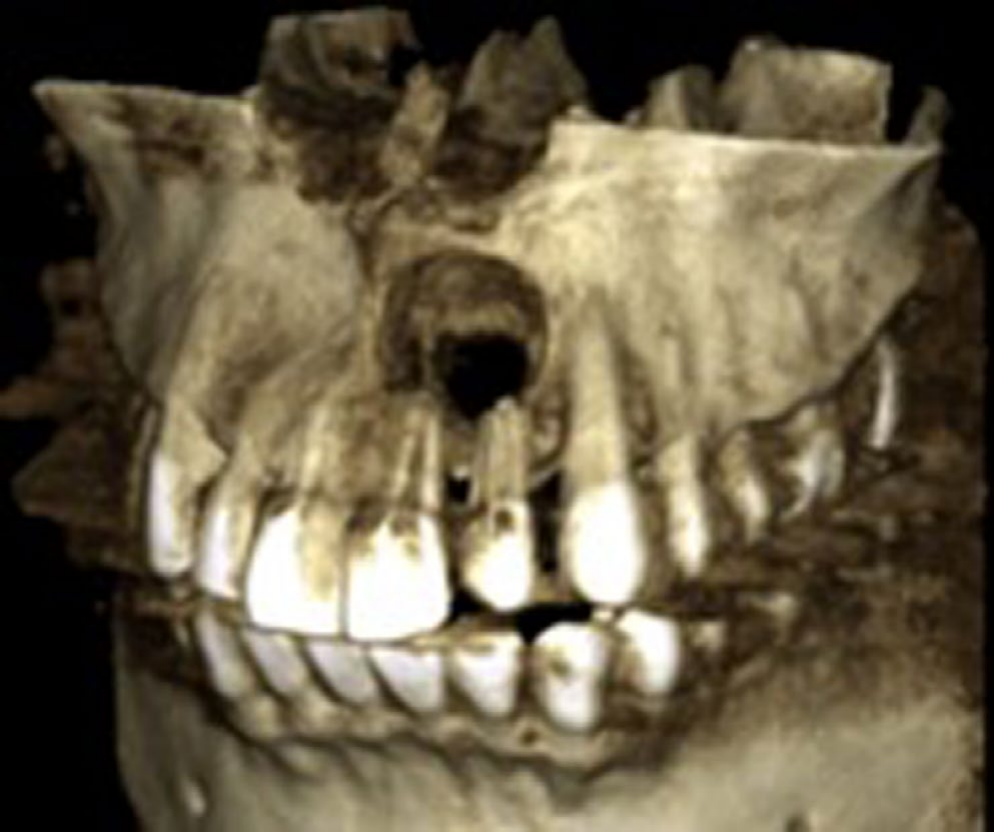
Cone Beam Computed Tomography reconstruction showed bone resorption at the apical of upper left central incisor and upper left lateral incisor with “tunnel” appearance

An intraoral periapical radiograph revealed a well‐defined unilocular rounded periapical radiolucency at the apices of the upper left central incisor and upper left lateral incisor (Figure 2). Cone Beam Computed Tomography (CBCT) revealed significant bone destruction including total volume of 552.04 mm^3^ (width of 10.56, height 8.00, and depth of 12.48) (Figure 3). The differential diagnosis was (a) periapical granuloma (b) radicular cyst and (c) chronic apical periodontitis. After explaining the treatment options, the patient agreed to undergo root canal treatment followed by surgical intervention for lesion via enucleation and to restore the defective bone.

## 3 CLINICAL PROCEDURES AND OUTCOME

**Figure 4:**
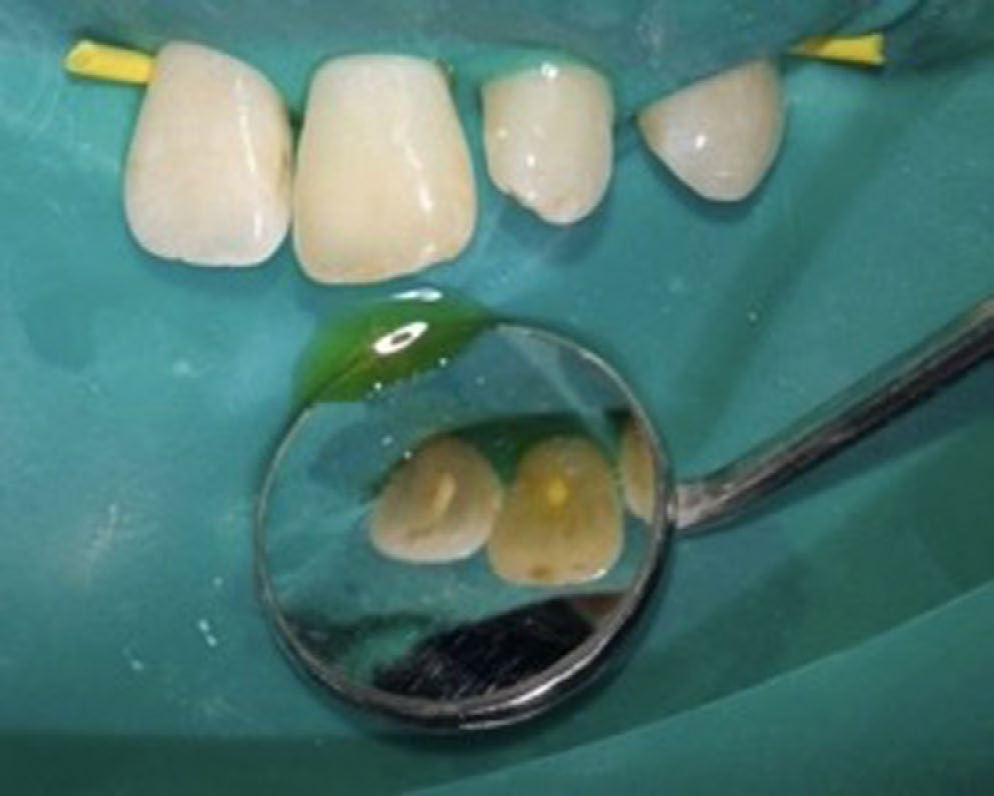
Multiple teeth isolation and clear fluid from the canal of upper left central incisor during root canal procedure

Verbal and written consent was obtained from the patient. Root canal treatment was completed on the upper right central incisor, upper left central incisor, and upper left lateral incisor. A dental operating microscope (Zeiss OPMI Ò pico, Germany) was used to enhance illumination and magnification, allowing the distinction of different dentin colors and facilitating the location of calcified dentine that obliterated the original canals. The obliterated canal of upper right central incisor and upper left lateral incisor was negotiated with 17% EDTA (MD‐Cleanser, Meta Biomed) solution and 19% EDTA (MD‐ChelCream, MetaBiomed) gel. Some clear fluid oozed out from the canal of the upper left central incisor (Figure 4). The canals were rinsed with sodium hypochlorite 2.5% (HUSM Pharmacy) and were prepared to the corrected working length with Pro‐Taper Universal hand files (Dentsply Maillefer) up to size F3 for both upper right central incisor and upper left central incisor. While size F2 preparations were prepared for the upper left lateral incisor. Intracanal medicament of nonsetting Pulpdent™ for the upper left lateral incisor. Intracanal medicament of nonsetting Pulpdent™ paste with 40% calcium hydroxide in an aqueous cellulose paste (Pulpdent Corporation) was placed in all canals. All the access cavities were temporarily restored with Fuji IX glass ionomer cement (GC International). The patient was recalled after a month and the teeth had no sign or symptoms of disease, and the swelling was slightly reduced in size.

**Figure 5:**
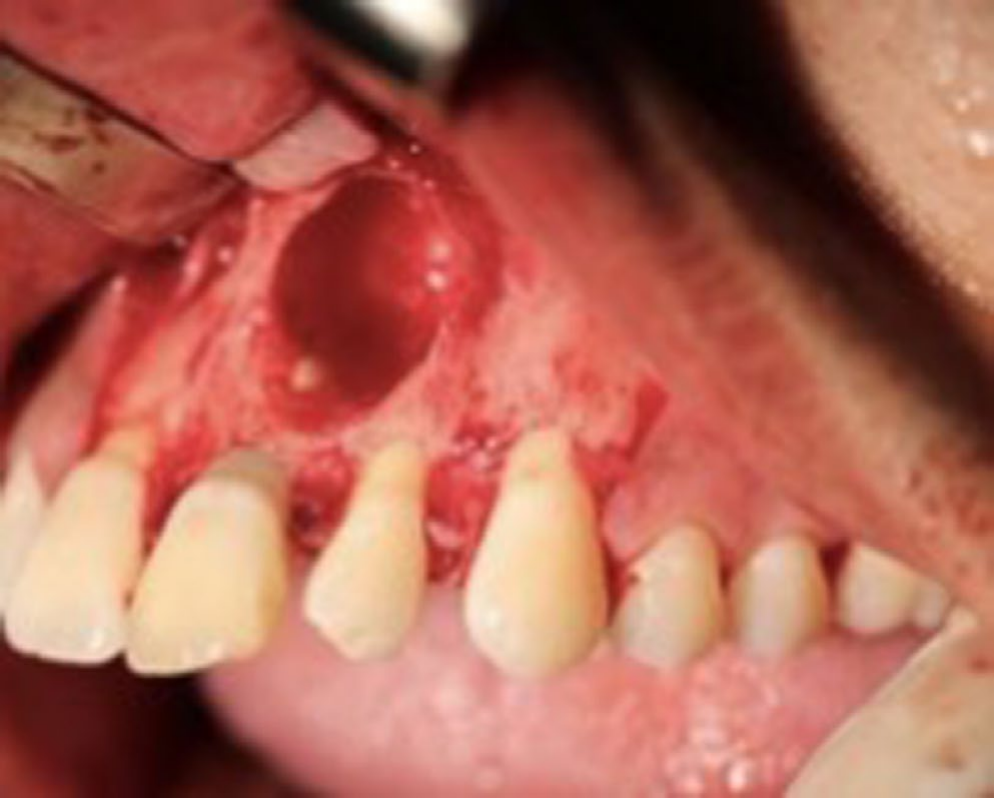
Circumferential area of bony defect was observed during the periapical surgical procedure

**Figure 6:**
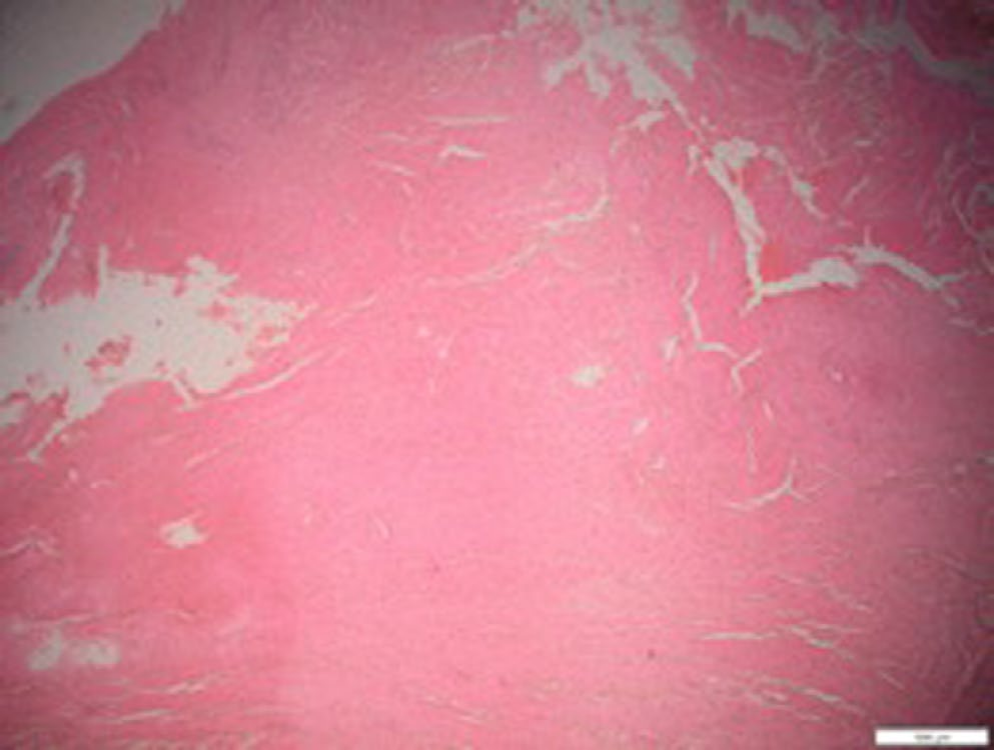
Microscopic appearance (10×) with nonkeratinized stratified squamous epithelium with evidence of multiple inflammatory cells in the fibrous connective tissue stroma

The root canal filling was performed using a single cone obturation followed by heat compaction. Gutta‐percha Pro‐Taper (F3) (Dentsply Maillefer) was used for both upper right and upper left central incisors. While gutta‐percha Pro‐Taper (F2) (Dentsply Maillefer) was for upper left lateral incisor. AH plus root canal sealer paste (Dentsply Maillefer) was delivered into all the canals using size 1 hand lentilo‐spiral (Dentsply Maillefer) until the prepared working length. The selected gutta‐percha was then inserted into each canal and compacted with a root canal plugger (Medesy). The excess gutta‐percha was removed to the level of cemento‐enamel junction and all the access cavity were cleaned from the debris and dirt using the cotton pellet. The access cavity for the teeth was finally restored with tooth colored restoration Zmack universal microhybrid composite (Zhermack SpA). The patient was recalled after 1 week and periapical surgery was performed. The operating site was anesthetized with 2% mepivacaine (Scandonestâ2% L, Septodont) with epinephrine (1:100 000). A sulcular incision from the region of mesial upper right lateral incisor to upper left canine was performed, and a mucoperiosteal flap was raised (Figure 5). The lesion was enucleated, and an apicectomy was performed by cutting 3 mm length from the apical root tip. Root‐end filling of Mineral Trioxide Aggregate (ProRoot^®^MTA, DenstplySirona) was placed to a 1 mm depth inside the apical space after removal of the gutta‐percha. Freeze‐dried cancellous Osteo‐LEMB bone graft (HUSM Tissue Bank) was placed at the bony defect to encourage the growth of key surrounding tissue. Flap closure was carried out with Dafilon 5.0^®^ (Aesculap), and the specimen was sent for histopathological examination. Ibuprofen (Biocare Group) 600 mg orally was prescribed to be taken twice daily only when necessary to control the pain and discomfort. The patient was recalled after a week for review and the sutures were removed. He had no complaint and the soft tissue healing was uneventful. Histopathological examination revealed that, there was a cystic cavity lined by a thin 2‐3 cells thick, nonkeratinized stratified squamous epithelium with evidence of multiple inflammatory cells in the fibrous connective tissue stroma (Figure 6).

**Figure 7:**
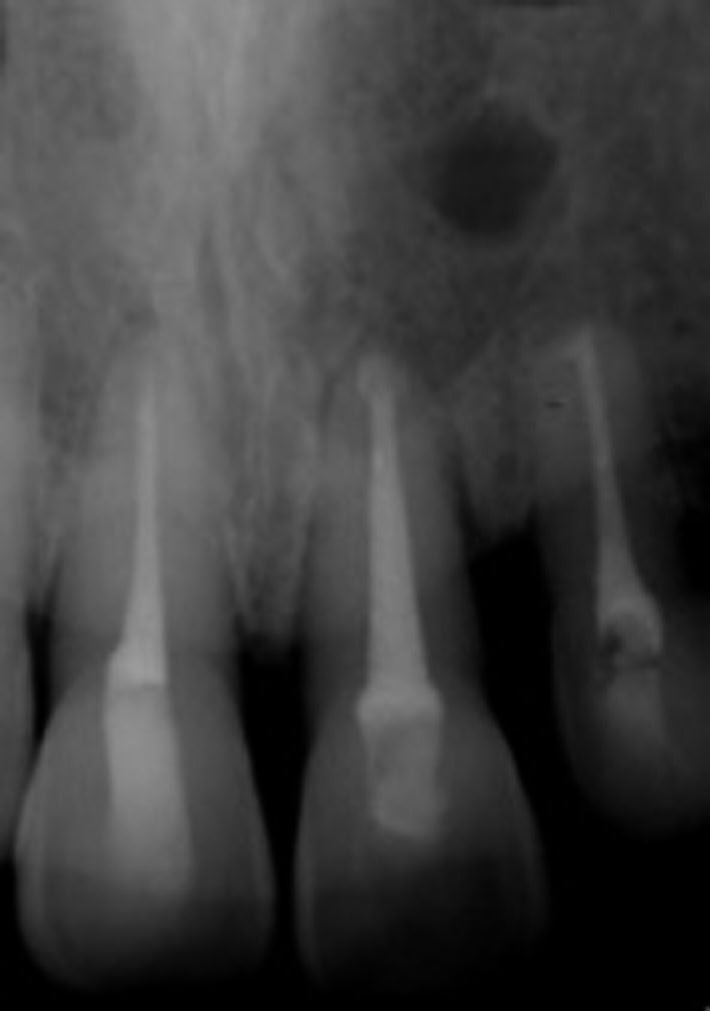
Periapical radiograph after 12 mo showing periapical healing, new bone formation and a small site of scar healing at apical of upper left lateral incisor

Based on the findings, the diagnosis was confirmed as a radicular cyst. Postoperative radiograph at the 1‐year follow‐up showed radiographical evidence of periapical repair and the presence of lamina dura. Though there was an evidence of radiographic bone formation, a small area of incomplete healing or healing by scar at the apical area of the upper left lateral incisor was noted. (Figure 7).

## 4 DISCUSSION

The patient presented with a history of dental trauma which caused a long‐standing pulpal necrosis and microbial infection. In the present case, it is speculated that, the development of the radicular cyst started once the epithelial cell rests of Malassez at the periapical area were stimulated from a continuous microbial infection from the pulp. The cells then continue to proliferate and multiply which later trigger an inflammatory process to release many IL‐17 cytokines and M1 macrophages, that mediate the attraction of neutrophils to the site. These ultimately promote the enlargement of the lesion.^3^, ^4^ There has been evidence of bone‐resorbing factors such as receptor activator of nuclear factor kappa‐B ligand and osteoprotegerin, which have a role in facilitating the cystic expansion.^5^ An average size of the radicular cyst can range between 0.5 to 1.5 cm.^6^ However, large radicular cysts with the measurement reaching up to 5.0‐10 cm have also been reported in various studies.^7^, ^8^, ^9^ As the size of the lesion increases, the surrounding alveolar bone usually becomes very thin due to subperiosteal bone deposition. Our patient presented with the condition where the cyst had completely eroded both buccal and palatal bone surfaces, which created a tunnel shaped appearance. Series of radiographic features may help in the assessment and examination of the oral condition. Surgical biopsy and histopathological evaluation remain the standard procedures for differentiating radicular cysts from other periapical pathologies.^10^ Histopathological analyses in a study reported that, nonkeratinized stratified squamous epithelial lining was found in 98.3% of the radicular cysts, while both mucoepidermoid epithelium and respiratory epithelium were found in 0.9% of the cases, followed by one case (0.4%) which revealed epithelial dysplasia of the epithelial lining.^11^

The treatment of choice may be determined by multiple factors such as lesion extension and its clinical characteristic, the involvement of important anatomical structures, patient preference and their systemic condition. Most radicular cysts can be managed with conventional root canal treatment without a surgical approach. However, an advantage of apical surgery is that, it enables excisional biopsy for histopathological assessment to confirm the diagnosis. Placing a bone graft following periapical surgery and cyst enucleation will accelerate bone formation at the defective area. The bone graft serves as a base for new bone formation and will slowly resorb to permit replacement by new bone. Bone grafts will also act as an osteoconductive material to stabilize blood clot and advance bone regeneration by providing a scaffold, thus enhancing the migration of osteoprogenitor cells.^12^, ^13^, ^14^ Our patient had received xenograft bone Osteo‐LEMB to restore the defective site since the technique was well accepted by him and does not contravene his faith and beliefs. Active and fast proliferation of the soft tissue from the buccal, lingual and palatal aspects may interfere with the bone ingrowth from approximal bone surfaces which result in a fibrous connective tissue core. The condition will be presented as an incomplete healing or scar tissue healing.^15^, ^16^ During 12 months postoperative review, our patient presented with small missing bone formation at the apical site of upper left lateral incisor. This could be manifestation of scar tissue healing or incomplete healing. The patient otherwise had no other complaints and the soft tissue healing was successful.

## 5 CONCLUSION

Conventional endodontic therapy followed by cyst enucleation proved to be successful for the management of a ‘tunnel’ radicular cyst. The used bone graft to fill the defective site had a positive outcome for the regenerative of new bone.

## CONFLICT OF INTEREST

None declared.

## AUTHOR CONTRIBUTIONS

NRNAG, NFAH, and MIK conceived and planned the case report. NRNAG and NFAH carried out the clinical procedure for this case report NRNAG and MIK planned and carried out the simulations. NRNAG and MIK took the lead in writing the manuscript and preparing the first and final draft. All authors provided critical feedback and helped shape the research, analysis and manuscript.
